# Point of care aspergillus testing in intensive care patients

**DOI:** 10.1186/s13054-020-03367-7

**Published:** 2020-11-10

**Authors:** Toine Mercier, Albert Dunbar, Vincent Veldhuizen, Michelle Holtappels, Alexander Schauwvlieghe, Johan Maertens, Bart Rijnders, Joost Wauters

**Affiliations:** 1 Department of Microbiology, Immunology and Transplantation, KU Leuven, Leuven, Belgium; 2 Department of Hematology, University Hospitals Leuven, Leuven, Belgium; 3 Department of Internal Medicine, Infectious Diseases, Erasmus University Medical Center, Rotterdam, The Netherlands; 4 Department of Hematology, University Hospitals Ghent, Ghent, Belgium; 5 Department of Intensive Care Medicine, University Hospitals Leuven, Leuven, Belgium

**Keywords:** Invasive aspergillosis, Diagnosis, Lateral flow assay, Galactomannan

## Abstract

**Background:**

Invasive pulmonary aspergillosis (IPA) is an increasingly recognized complication in intensive care unit (ICU) patients, especially those with influenza, cirrhosis, chronic obstructive pulmonary disease, and other diseases. The diagnosis can be challenging, especially in the ICU, where clinical symptoms as well as imaging are mostly nonspecific. Recently, *Aspergillus* lateral flow tests were developed to decrease the time to diagnosis of IPA. Several studies have shown promising results in bronchoalveolar lavage fluid (BALf) from hematology patients. We therefore evaluated a new lateral flow test for IPA in ICU patients.

**Methods:**

Using left-over BALf from adult ICU patients in two university hospitals, we studied the performance of the *Aspergillus* galactomannan lateral flow assay (LFA) by IMMY (Norman, OK, USA). Patients were classified according to the 2008 EORTC-MSG definitions, the AspICU criteria, and the modified AspICU criteria, which incorporate galactomannan results. These internationally recognized consensus definitions for the diagnosis of IPA incorporate patient characteristics, microbiology and radiology. The LFA was read out visually and with a digital reader by researchers blinded to the final clinical diagnosis and IPA classification.

**Results:**

We included 178 patients, of which 55 were classified as cases (6 cases of proven and 26 cases of probable IPA according to the EORTC-MSG definitions, and an additional 23 cases according to the modified AspICU criteria). Depending on the definitions used, the sensitivity of the LFA was 0.88–0.94, the specificity was 0.81, and the area under the ROC curve 0.90–0.94, indicating good overall test performance.

**Conclusions:**

In ICU patients, the LFA performed well on BALf and can be used as a rapid screening test while waiting for other microbiological results.

## Introduction

Invasive pulmonary aspergillosis (IPA) is increasingly being recognized as an important complication in intensive care unit (ICU) patients, especially in patients with severe influenza infection, liver cirrhosis, or chronic obstructive pulmonary disease (COPD) [1–3]. More recently, patients with coronavirus viral disease 2019 (COVID-19) also appeared to be at an increased risk for IPA [4, 5]. However, the diagnosis can be challenging and demands a specific workup as radiologic signs are less typical in non-neutropenic patients, and clinical signs are non-specific [3]. This becomes apparent in autopsy series from ICU patients, where IPA remains one of the most commonly missed diagnoses [6, 7]. A recent retrospective study found a post-mortem diagnosis of IPA in 25 (2.8%) of the 893 autopsies performed between 1991 and 2016 in critically ill patients [7]. As a delay in antifungal treatment is correlated with a significantly higher mortality, these diagnostic difficulties can lead to worse outcomes [10].

The only indisputable way of making a “proven” diagnosis of IPA is through a biopsy showing *Aspergillus* hyphae (either pre- or post-mortem). Unfortunately, a biopsy is not feasible in the majority of ICU patients. Because of these diagnostic difficulties, international consensus definitions for the diagnosis of IPA for research purposes were developed, such as the European Organization for Research and Treatment of Cancer/Invasive Fungal Infections Cooperative Group and the National Institute of Allergy and Infectious Diseases Mycoses Study Group (EORTC/MSG) definitions. The use of consensus definitions (in the absence of a useable gold standard) has been accepted by regulatory body such as the FDA and EMA, both for treatment studies as well as for diagnostic studies. However, these definitions have mainly been developed for use in immunocompromised patients [8]. Indeed, in an evaluation of a clinical algorithm for the diagnosis of IPA developed specifically for use in ICU patients, 84% of cases could not be classified using the EORTC/MSG definitions [9]. For this reason, disease definitions targeting ICU populations were recently developed by Blot et al. in the AspICU study, and later modified by Schauwvlieghe et al. [1, 9]. The original AspICU criteria, developed by Blot et al., were modified as the original criteria require a positive culture for *Aspergillus* as entry criterion, even though cultures are negative in a large majority of cases [10]. In summary, the modified AspICU criteria eliminate the requirement of a positive culture and incorporate galactomannan (GM) as a sufficient mycological criterion, and allow the inclusion of patients with high-risk diseases such as influenza or COPD, that do not have any “classical” host factors such as neutropenia. Detection of GM, an antigen that is present in the cell wall of *Aspergillus*, by the Platelia™ enzyme immunoassay, is widely used as a diagnostic tool in IPA. The sensitivity and specificity of GM detection in bronchoalveolar lavage fluid (BALf) ranges between 0.61–0.92, and 0.89–0.98, respectively [11–13]. This makes it more sensitive than direct microscopy, fungal culture, or serum GM detection [14]. The variation in performance depends on the use of different cut-off values, the population being tested, or the case definition that is used in the study [11–13]. It is important to note that these diagnostic characteristics were mainly derived from studies that almost exclusively included patients with an underlying hematological disease. In the most informative study to date in which 26 cases of biopsy proven IPA were included, the sensitivity and specificity of BALf GM ≥ 0.5 were 0.88 and 0.87, respectively [14].

However, GM detection using the Platelia™ assay is not always available on-site and often has a long turnaround time to decrease the per-test cost by batching tests, increasing the diagnostic delay [15]. A fast, single sample test could therefore decrease diagnostic delay. Furthermore, a single sample test would make the handling of samples from patients with a highly contagious disease (such as COVID-19) easier.

Recently, two lateral flow tests have been developed which could facilitate a rapid diagnosis of IPA on single samples. These are the AspLFD lateral flow device (LFD) by OLM Diagnostics (Newcastle upon Tyne, UK) and the sōna Aspergillus galactomannan lateral flow assay (LFA) by IMMY (Norman, OK, USA). These assays can be performed similarly to the widely known pregnancy tests: after application of the sample to the sample site, the appearance of a line at the control site indicates a valid test, and the appearance of a line at the test site indicates a positive test result. The results from both lateral flow tests are available within 15 min to 1 h after sampling, depending on the test and sample used.

A comparative study of both tests in BALf from hematological patients showed that the LFA had a significantly higher sensitivity than the LFD (0.83 versus 0.69) [17]. To date, only a single study in 133 ICU patients evaluated a prototype version of the LFD, showing a sensitivity and specificity of 0.80 and 0.81, respectively [18]. No studies have evaluated the LFA or the commercialized version of the LFD in ICU patients, nor are there any comparative studies in this population. Based on the superior results of the LFA in previous studies, and because of the lack of data in ICU patients—which are often significantly different from hematology patients—we performed a clinical study to evaluate the LFA as a rapid diagnostic test in ICU patients at risk for IPA.

## Materials and methods

We retrospectively collected BALf samples from patients admitted to the ICU in two academic centers (Erasmus University Medical Center, Rotterdam, The Netherlands, and University Hospitals Leuven, Leuven, Belgium) from between 2013 and 2019. Patients could be enrolled if they (1) were 18 years of age or older, (2) were admitted to the ICU, and (3) had sufficient BALf remaining stored at − 20 °C. In order to minimize uncertainty about the presence or absence of IPA, we excluded patients (1) with EORTC/MSG defined possible invasive fungal disease, (2) with BALf GM ≥ 0.5 and < 1.0, (3) with positive mycological findings (such as GM or culture) that did not receive mold-active antifungal therapy and survived, (4) with probable or putative IPA in whom subsequent autopsy could not reveal any sign of IPA, and (5) that had received systemic mold-active treatment ≥ 72 h before BALf sampling. For each patient, we collected the following data: gender, age, weight, primary underlying disease, mycological results (fungal cultures, direct microscopy, and histopathology), autopsy results (if performed), absolute neutrophil count, and survival after diagnosis. Due to the retrospective nature of this study on remaining fractions of previously collected samples for diagnostic purposes, the need for informed consent was waived.

### Case definitions

Cases were classified according to the 2008 revised EORTC/MSG definitions [8], the AspICU definitions as published by Blot et al. [9], and the modified AspICU definitions as published by Schauwvlieghe et al. [1]. For the determination of the diagnostic characteristics, true positives (“cases”) were defined as those with proven or probable IPA (for the EORTC/MSG definitions), or those fulfilling the AspICU or modified AspICU definitions. True negatives (“controls”) had, as defined by the inclusion and exclusion criteria, no signs of *Aspergillus* in mycological tests (negative culture, microscopy, and GM) or at autopsy, did not receive systemic mold-active therapy, and did not have possible IPA.

### BALf testing

All frozen BALf samples were defrosted at room temperature and vortexed briefly. Galactomannan was tested using the Platelia™ *Aspergillus* enzyme immunoassay (Bio-Rad, Marnes-la-Coquette, France) in accordance with the manufacturer’s instructions. The *Aspergillus* galactomannan lateral flow assay (IMMY, Norman, Oklahoma, USA) was performed in accordance with the manufacturer’s instructions. Visual readout was performed by a single, experienced researcher, blinded to the final diagnosis of the patient. The LFA result was confirmed objectively using a digital reader (Cube reader, Chembio Diagnostics GmbH, Berlin, Germany), with a result of ≥ 0.5 considered as positive. The results returned by the reader are a dimensionless value, calibrated by the manufacturer to mimic the results from the Platelia™ assay, with positive results fixed at a cutoff at 0.5. Unless otherwise stated, all analyses in this study use the digital result of the test and not the visual result. The LFA was provided to us by IMMY; the manufacturer had no role in the design of this study, its execution, analysis, interpretation of the data, or decision to publish.

### Statistical analysis

To obtain the ability to calculate sensitivity and specificity with a maximum of 12% width of the 95% confidence interval, at 80% power and at the 95% confidence interval, we used the summary data previously published by Mercier et al. [17] and Jenks et al. [19], and calculated appropriate sample sizes using the method as described by Buderer et al. [20].

Based on the reported pooled sensitivity of 73% and pooled specificity of 90%, with an expected prevalence of 30% in patients undergoing bronchoscopy for suspected IPA, we estimated a total of 175 patients (122 cases + 53 controls). We determined the sensitivity, specificity, positive and negative predictive values (assuming a final prevalence of IPA of 30% in patients undergoing bronchoscopy for suspected IPA) with their respective 95% confidence intervals (CI’s) according to each classification system (EORTC/MSG, AspICU and modified AspICU). Cox regression was used to analyze survival as a function of LFA intensity or positivity. For comparison between the serum GM and the LFA, we used McNemar’s test for pairwise observations. For within group comparisons (e.g. neutropenic status or centrum effect), we used Fisher’s exact test. Statistical analysis was performed using R v3.6.1 (R Foundation for Statistical Computing, Vienna, Austria).

## Results

We included a total of 178 patients in our study. Patient characteristics are shown in Table 1. BALf was stored for a median of 118.7 weeks before testing (interquartile range 32.75–227). All samples had a volume of 600 µL or more.

**Table 1:** Patient characteristics

	Case	Control	p
*n*	55	123	
Center = Rotterdam (%)	22 (40.0)	54 (43.9)	0.747
Age, years (median [IQR])	63 [56, 68]	57 [46, 66]	0.073
Male gender (%)	34 (61.8)	66 (53.7)	0.395
Weight, kg (median [IQR])	70 [60, 84]	70 [62, 85]	0.910
Underlying disease (%)			0.355
Pulmonary disease	22 (40.0)	59 (52.2)	
Hematologic malignancy	9 (16.4)	10 (8.8)	
Heart disease	4 (7.3)	10 (8.8)	
Liver disease	3 (5.5)	5 (4.4)	
Gastrointestinal disease	3 (5.5)	2 (1.8)	
Other malignancy	2 (3.6)	9 (8.0)	
Other	12 (21.8)	18 (15.9)	
Neutropenia (%)	8 (17.0)	7 (5.7)	0.094
Influenza (%)	17 (30.9)	47 (38.2)	0.442
COPD (%)	6 (10.9)	15 (12.2)	1.000
Positive culture (%)	28 (50.9)		
Positive microscopy (%)	4 (7.3)		
BALf GM (median [IQR])	4.80 [2.73, 5.68]		

*IQR* interquartile range, *BALf GM* bronchoalveolar lavage fluid galactomannan, *COPD* chronic obstructive pulmonary disease

Using the EORTC/MSG definitions, we identified 6 cases of proven IPA and 26 cases of probable IPA. Using the modified AspICU definitions, we identified an additional 23 cases fulfilling the criteria, for a total of 55 cases. Using the AspICU definitions (which has a positive culture for *A. fumigatus* as entry criterion for probable or putative IPA), we identified 6 cases of proven IPA and 12 cases of putative IPA. Of these 55 cases, 51 were treated using mold-active antifungal drugs while at the ICU. The reason for not initiating therapy (e.g. because of missed diagnosis, death, starting of therapy after leaving the ICU, stopping of curative therapy, or an alternative diagnosis) was not recorded in this study.

**Fig. 1:**
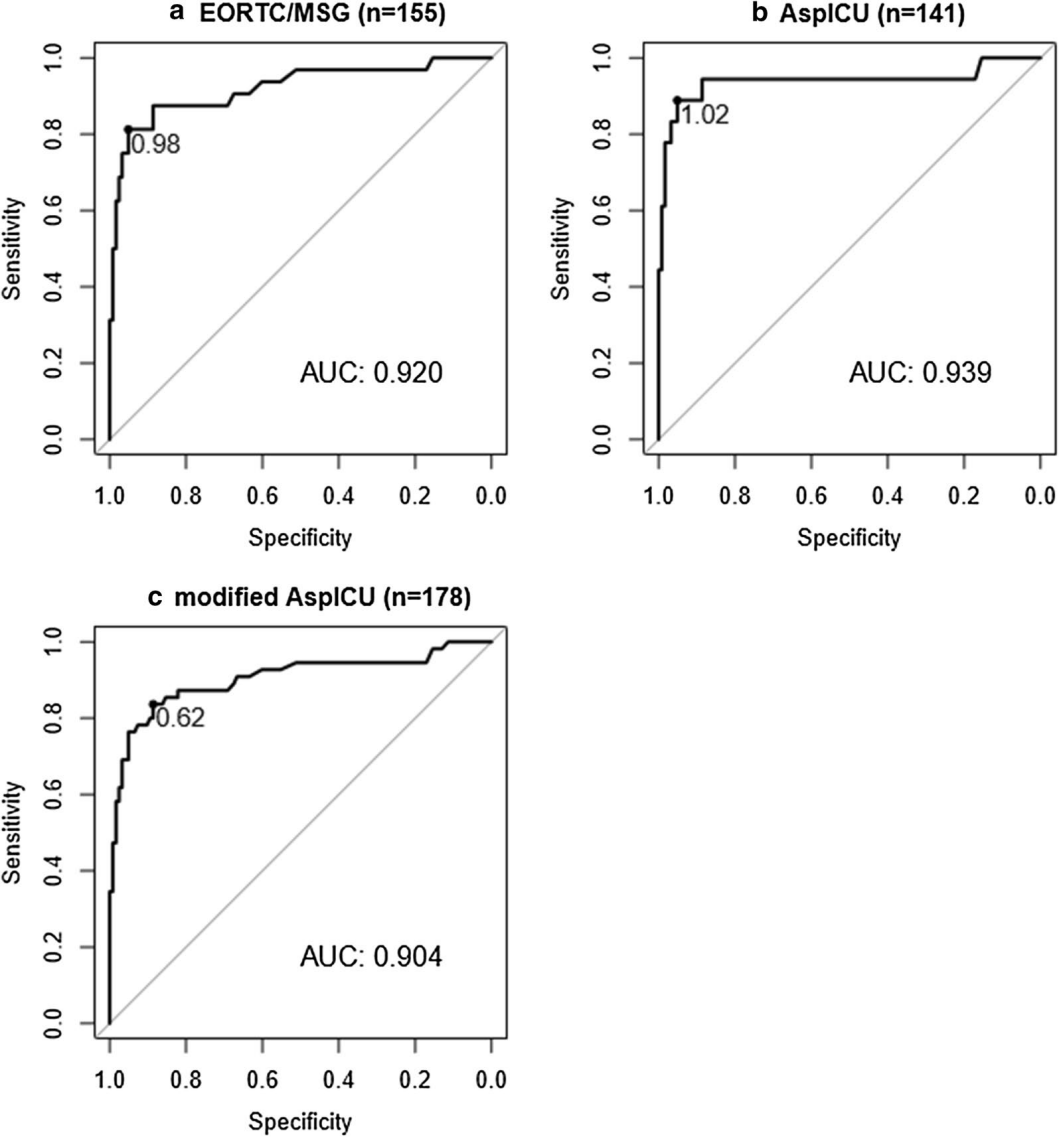
ROC curves for digital readout of the lateral flow assay. The dot indicates the cutoff with the highest Youden index

**Fig. 2:**
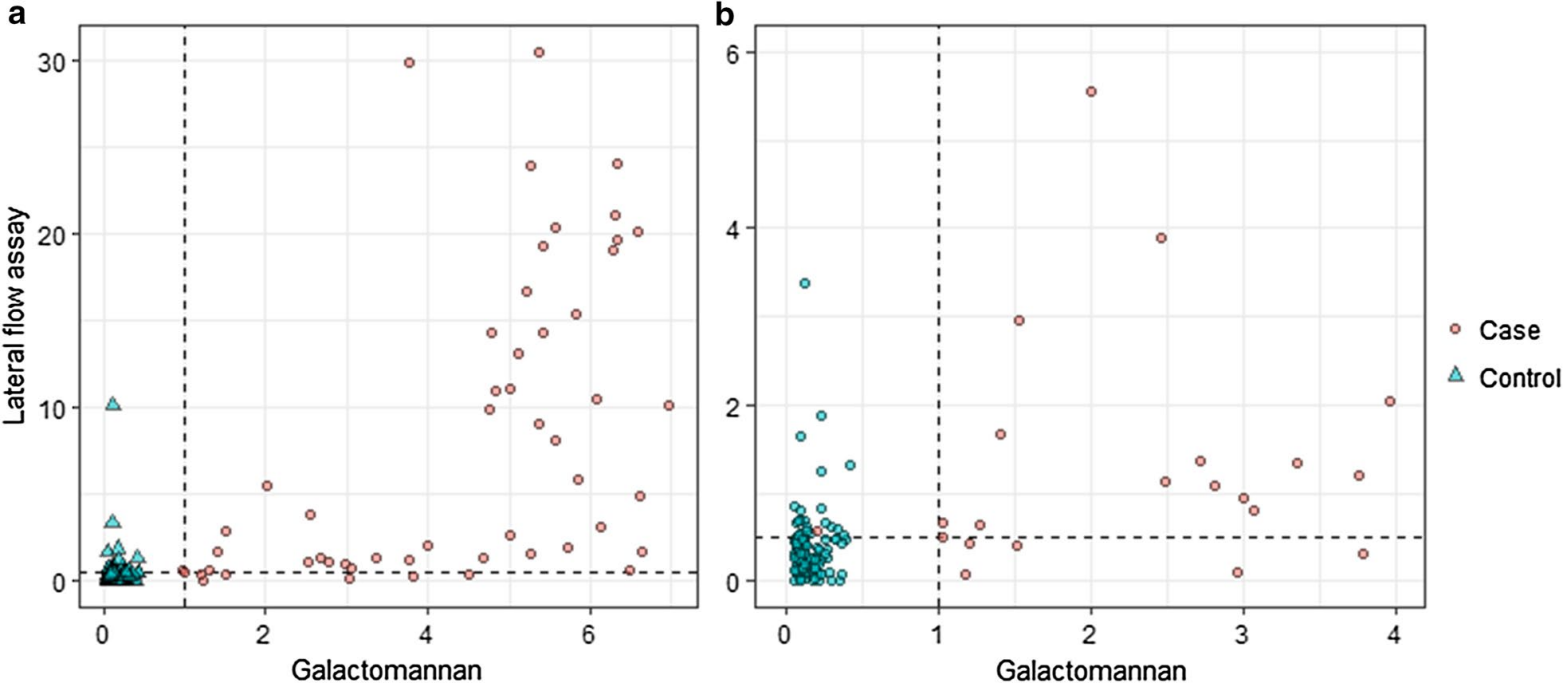
Correlation of galactomannan and lateral flow assay. Pearson’s *r* = 0.719 (*p* < 0.001). **a** Overview of all subjects. **b** Zoomed in detail

The diagnostic characteristics of the LFA for the different disease classifications are shown in Table 2 (digital readout) and Table 3 (visual readout). Using digital readout significantly increased the sensitivity and negative predictive value compared to visual readout in the modified AspICU group (*p* = 0.008 and 0.044, respectively). Conversely, the specificity and positive predictive value were significantly lower when using digital readout (*p* < 0.001 for both). The ROC curves for all three classifications are shown in Fig. 1. The correlation between GM levels and LFA results is shown in Fig. 2.

**Table 2:** Diagnostic characteristics (including their 95% confidence interval) of digital readout of the lateral flow assay for the different disease definitions

	Classification	Sensitivity	Specificity	Negative predictive value	Positive predictive value
All Cases	EORTC/MSG (*n* = 155)	0.88 (0.71–0.96)	0.81 (0.73–0.88)	0.94 (0.86–0.97)	0.67 (0.58–0.75)
	AspICU (*n* = 141)	0.94 (0.73–1.00)	0.81 (0.73–0.88)	0.97 (0.84–1.00)	0.68 (0.60–0.76)
	modified AspICU (*n* = 178)	0.87 (0.76–0.95)	0.81 (0.73–0.88)	0.94 (0.88–0.97)	0.67 (0.58–0.75)
Criteria excluding galactomannan	EORTC/MSG (*n* = 140)	1.00 (0.80–1.00)	0.81 (0.73–0.88)	0.96 (0.84–0.99)	0.67 (0.58–0.74)
	modified AspICU (*n* = 152)	0.97 (0.82–1.00)	0.81 (0.73–0.88)	0.98 (0.89–1.00)	0.69 (0.60–0.76)

**Table 3:** Diagnostic characteristics (including their 95% confidence interval) of visual readout of the lateral flow assay for the different disease definitions

	Classification	Sensitivity	Specificity	Negative predictive value	Positive predictive value
All cases	EORTC/MSG (*n* = 155)	0.81 (0.64–0.93)	0.95 (0.90–0.98)	0.92 (0.85–0.96)	0.88 (0.76–0.94)
	AspICU (*n* = 141)	0.89 (0.65–0.99)	0.95 (0.90–0.98)	0.95 (0.84–0.99)	0.89 (0.78–0.95)
	modified AspICU (*n* = 178)	0.75 (0.61–0.85)	0.95 (0.90–0.98)	0.90 (0.85–0.93)	0.87 (0.75–0.94)
Criteria excluding galactomannan	EORTC/MSG (*n* = 140)	0.94 (0.71–1.00)	0.95 (0.90–0.98)	0.97 (0.85–1.00)	0.89 (0.79–0.95)
	Modified AspICU (*n* = 152)	0.86 (0.68–0.96)	0.95 (0.90–0.98)	0.94 (0.87–0.98)	0.88 (0.77–0.94)

In patients in which this was performed, serum GM was significantly less sensitive than the LFA (0.88 vs 0.33, *p* < 0.001), while serum GM was more specific (1.00 vs 0.89, *p* = 0.014).

The sensitivity was not significantly different in neutropenic patients vs non-neutropenic patients (0.75 vs 0.89, *p* = 0.267), in patients with hematologic malignancy (0.67 vs 0.91, *p* = 0.078), in patients with COPD (1.00 vs 0.86, *p* = 1.000), in patients with influenza (0.94 vs 0.84, *p* = 0.416) or in patients receiving antifungal prophylaxis > 72 h before sampling (0.78 vs 0.89, *p* = 0.321). We could not identify any centrum effect on sensitivity or specificity (*p* = 0.491 and *p* = 1.000, respectively).

**Fig. 3:**
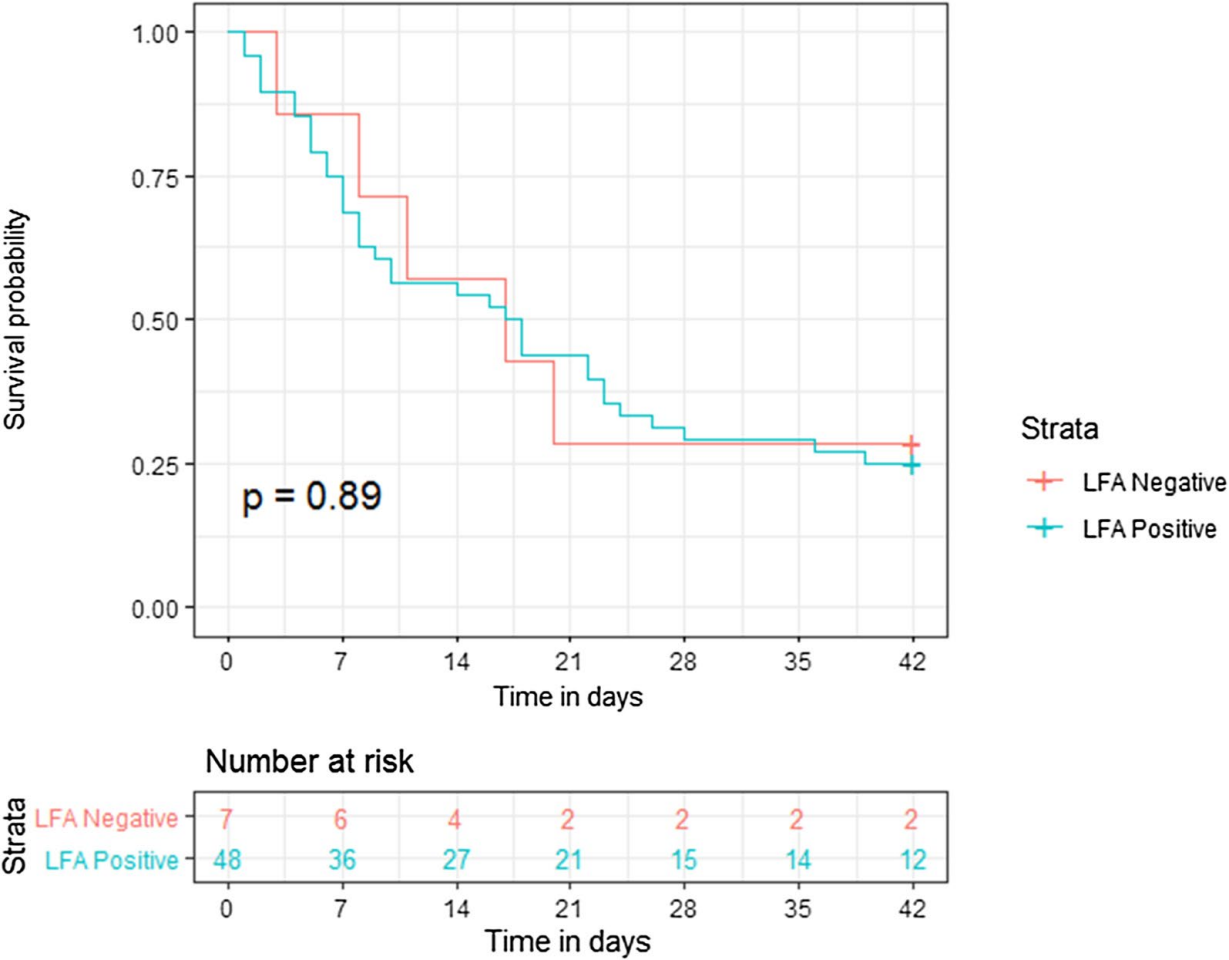
Kaplan–Meier survival plot of modified AspICU cases of invasive aspergillosis, stratified by LFA positivity

The LFA in BALf was not predictive of survival (Fig. 3). This effect remained not significant after correcting for age, neutropenic status, presence of influenza or COPD, and underlying disease, either when used as a binary predictor (*p* = 0.290) or as a continuous predictor (*p* = 0.208).

## Discussion

This is the first study to evaluate the performance of the LFA in ICU patients. This test could allow a faster diagnosis of IPA to be made in ICU patients. Because obtaining a “proven” diagnosis of IPA can often be challenging in the ICU population, we also included patients with so-called probable or putative disease. These patients have a very high likelihood of having IPA, although there will no doubt be patients that are wrongly classified as having (or as not having) IPA. For this reason, we classified each patient according to the three most widely used disease definitions, to avoid bias and to allow comparison with other diagnostic studies. These definitions are used internationally in both epidemiologic, diagnostic and therapeutic studies and allow the creation of homogenous diagnostic groups across different studies. In our study of 178 patients, of which 55 were classified as cases according to the modified AspICU criteria (which include a positive culture result for *Aspergillus*, or GM ≥ 1.0 as a mycologic criterion), and 123 as control subjects without any microbiological evidence of *Aspergillus*. The modified AspICU criteria, developed by Schauwvlieghe et al*.*, were used to identify patients admitted to the ICU with IPA, but not fulfilling the EORTC/MSG criteria.

In our total study population, we found a good sensitivity of 0.88 and specificity of 0.81. Although 17% of the cases were neutropenic at time of diagnosis, the sensitivity of the LFA was not significantly higher in neutropenic patients than in non-neutropenic patients. When restricting the cases to only those classified as cases by the original AspICU criteria, the sensitivity even increased to 0.94. A possible explanation for the increased sensitivity in this subgroup is that by definition, all of these cases had a positive fungal culture result. Previous studies have indeed also shown that a positive culture result is associated with higher LFA intensities [17]. Interestingly, the sensitivity in our study was significantly higher than that found in a small study on 26 non-neutropenic patients (five of which were ICU patients) with IPA according to the original AspICU criteria [21]. It is not clear what could explain this difference. When looking purely at cases that were classifiable according to the EORTC/MSG definitions in our population, our results were similar to a recent study in BALf from hematology patients, which found a sensitivity of 0.83 (versus 0.88 in this ICU population) and a specificity of 0.87 (versus 0.81 in this ICU population). As the LFA was designed to detect GM, and GM is a mycologic criterion, there is a risk of incorporation bias. Furthermore, there was a strong correlation between GM levels as determined by the Platelia™ assay and LFA intensity. We therefore analyzed the performance in a subgroup where GM was excluded from the criteria. In this subgroup, we found a similar or even improved performance of the LFA, indicating that incorporation bias is unlikely.

Initially, there were concerns over visual interpretation of the test due to the presence of a weak line at the test site [17]. This was overcome by using a digital reader, improving both the sensitivity and specificity of the assay by eliminating uncertainty over low positive results. Since this initial study, the manufacturer of the LFA has released a compact digital reader for use with the test strip, simplifying and standardizing readout, eliminating inter-observer variability and allowing exact quantification of the test result. Indeed, in this study, this manufacturer-supplied digital reader increased the sensitivity and negative predictive value. However, the specificity and positive predictive value were decreased when using this reader, indicating that in our study, the use of reader functions more like a lower test cutoff, rather than increasing the overall accuracy.

Our study has several limitations. All samples had been frozen until further analysis. However, in a previous study, we found the GM value to be consistent when a new GM measurement was performed. Mold-active prophylaxis was a strict exclusion criterion for the control group to maximize the likelihood of patients not having IPA, as prophylaxis can lead to false negative microbiologic tests [22]. This exclusion criterion was not used in the case group, which could lead to bias. However, we did not notice a significant relation between false negativity and mold-active prophylaxis.

After designing, performing and analyzing this study, an update to the EORTC/MSG definitions was published in December 2019 [23]. In this update, an additional radiologic sign was added and the cutoff values were defined more precisely. This means that patients that fulfill the 2008 version of these definitions will also fulfill the 2019 version, provided BALf GM is ≥ 1.0, which is the case in our study, by design. However, it is possible that some patients that are now classified as ‘unclassifiable’ in our study, based on the 2019 version, would become classified as having probable IPA using the 2019 version. Furthermore, consensus definitions on influenza-associated pulmonary aspergillosis (IAPA) were published in June 2020 [24]. In our study, 31% of the cases had a positive influenza test. However, all of these cases fulfilled the modified AspICU criteria, thereby also fulfilling these new IAPA definitions.

According to the package insert, cross-reactivity can occur based on positive results from culture filtrate from *Candida* spp., *Coccidioides* spp., *Paracoccidiodes brasiliensis*, and *Histoplasma* spp. Whether this in vitro cross-reactivity is clinically relevant, is unclear. One retrospective study found positive LFA results in BALf and sputum that grew *Scedosporium* spp., *Fusarium* spp., *Saccharomyces cerevisiae*, *Candida parapsilosis*, and *Geotrichium* spp. in fungal culture [16]. It is not clear if this constitutes true cross-reactivity or rather undetected co-infection with *Aspergillus* or another closely related fungus.

As this is the first study of this diagnostic test in ICU patients, we wanted to differentiate patients with IPA as best as possible from those without IPA. We therefore excluded patients with a high degree of diagnostic uncertainty, as we would not be able to interpret the LFA results from these patients unambiguously (i.e. is a negative LFA in a patient with possible disease a false negative, or does this indicate that the pulmonary lesion is caused by another disease, in which case the LFA result is actually a true negative). Once the diagnostic accuracy of this test is more clearly defined in different studies, patients with possible disease can be included as well in an effort to clarify if these patients really do have an invasive fungal infection or not.

In conclusion, the sōna Aspergillus galactomannan LFA on BALf appears to be a good diagnostic aid for IPA in ICU patients. This fast assay can be particularly useful in centers with a long turnaround time for more conventional tests such as the Platelia™ GM assay. Independent of the disease definitions used, the LFA provided sufficiently reliable results to be used as a rapid diagnostic test awaiting further confirmatory tests such as GM, PCR or culture. The latter can strengthen or reject the LFA test result and thereby confirm the presence or absence of Aspergillus. The LFA could aid in quick clinical decisions in ICU patients which eventually may improve survival of patients with an invasive *Aspergillus* infection.

## Conclusions

In ICU patients, the LFA performed well on BALf and can be used as a rapid screening test while waiting for other microbiological results.

## Data Availability

The datasets used and/or analyzed during the current study are available from the corresponding author on reasonable request.

## Abbreviations

BALf: Bronchoalveolar lavage fluid
COPD: Chronic obstructive pulmonary disease
COVID-19: Coronavirus viral disease 2019
EORTC/MSG: European Organization for Research and Treatment of Cancer/Invasive Fungal Infections Cooperative Group and the National Institute of Allergy and Infectious Diseases Mycoses Study Group
GM: Galactomannan
ICU: Intensive care unit
IPA: Invasive pulmonary aspergillosis
IQR: Interquartile range
LFA: IMMY galactomannan lateral flow assay
LFD: OLM Diagnostics AspLFD lateral flow device

## References

[1] Schauwvlieghe AFAD, Rijnders BJA, Philips N, Verwijs R, Vanderbeke L, Tienen CV (2018). Invasive aspergillosis in patients admitted to the intensive care unit with severe influenza: a retrospective cohort study. Lancet Respir Med.

[2] Meersseman W, Vandecasteele SJ, Wilmer A, Verbeken E, Peetermans WE, Van Wijngaerdert E (2004). Invasive aspergillosis in critically ill patients without malignancy. Am J Respir Crit Care Med.

[3] Taccone FS, Van den Abeele A-M, Bulpa P, Misset B, Meersseman W, Cardoso T (2015). Epidemiology of invasive aspergillosis in critically ill patients: clinical presentation, underlying conditions, and outcomes. Crit Care.

[4] Koehler P, Cornely OA, Böttiger BW, Dusse F, Eichenauer DA, Fuchs F (2020). COVID-19 associated pulmonary aspergillosis. Mycoses.

[5] Alanio A, Dellière S, Fodil S, Bretagne S, Mégarbane B (2020). Prevalence of putative invasive pulmonary aspergillosis in critically ill patients with COVID-19. Lancet Respir Med..

[6] Roosen J, Frans E, Wilmer A, Knockaert DC, Bobbaers H (2000). Comparison of premortem clinical diagnoses in critically iII patients and subsequent autopsy findings. Mayo Clin Proc.

[7] Tejerina EE, Abril E, Padilla R, Ruíz CR, Ballen A, Frutos-Vivar F (2019). Invasive aspergillosis in critically ill patients: an autopsy study. Mycoses.

[8] de Pauw B, Walsh TJ, Donnelly JP, Stevens DA, Edwards JE, Calandra T (2008). Revised Definitions of Invasive Fungal Disease from the European Organization for Research and Treatment of Cancer/Invasive Fungal Infections Cooperative Group and the National Institute of Allergy and Infectious Diseases Mycoses Study Group (EORTC/MSG) Consensus Group. Clin Infect Dis.

[9] Blot SI, Taccone FS, Van den Abeele A-M, Bulpa P, Meersseman W, Brusselaers N (2012). A clinical algorithm to diagnose invasive pulmonary aspergillosis in critically ill patients. Am J Respir Crit Care Med.

[10] Lamoth F, Calandra T (2017). Early diagnosis of invasive mould infections and disease. J Antimicrob Chemother.

[11] Zou M, Tang L, Zhao S, Zhao Z, Chen L, Chen P (2012). Systematic review and meta-analysis of detecting galactomannan in bronchoalveolar lavage fluid for diagnosing invasive aspergillosis. PLoS ONE.

[12] Guo Y-L, Chen Y-Q, Wang K, Qin S-M, Wu C, Kong J-L (2010). Accuracy of BAL galactomannan in diagnosing invasive aspergillosis: a bivariate metaanalysis and systematic review. Chest.

[13] Heng SC, Morrissey O, Chen SC-A, Thursky K, Manser RL, Nation RL (2015). Utility of bronchoalveolar lavage fluid galactomannan alone or in combination with PCR for the diagnosis of invasive aspergillosis in adult hematology patients: A systematic review and meta-analysis. Crit Rev Microbiol.

[14] Meersseman W, Lagrou K, Maertens J, Wilmer A, Hermans G, Vanderschueren S (2008). Galactomannan in bronchoalveolar lavage fluid: a tool for diagnosing aspergillosis in intensive care unit patients. Am J Respir Crit Care Med.

[15] Schauwvlieghe AFAD, de Jonge N, van Dijk K, Verweij PE, Brüggemann RJ, Biemond BJ (2018). The diagnosis and treatment of invasive aspergillosis in Dutch haematology units facing a rapidly increasing prevalence of azole-resistance. A nationwide survey and rationale for the DB-MSG 002 study protocol. Mycoses.

[16] Lass-Flörl C, Cascio GL, Nucci M, dos Santos MC, Colombo AL, Vossen M (2019). Respiratory specimens and the diagnostic accuracy of Aspergillus lateral flow assays (LFA-IMMY^TM^): real-life data from a multicentre study. Clin Microbiol Infect Elsevier.

[17] Mercier T, Dunbar A, de Kort E, Schauwvlieghe A, Reynders M, Guldentops E (2019). Lateral flow assays for diagnosing invasive pulmonary aspergillosis in adult hematology patients: a comparative multicenter study. Med Mycol..

[18] Eigl S, Prattes J, Lackner M, Willinger B, Spiess B, Reinwald M (2015). Multicenter evaluation of a lateral-flow device test for diagnosing invasive pulmonary aspergillosis in ICU patients. Crit Care Lond Engl.

[19] Jenks JD, Mehta SR, Taplitz R, Law N, Reed SL, Hoenigl M (2019). Bronchoalveolar lavage Aspergillus Galactomannan lateral flow assay versus Aspergillus-specific lateral flow device test for diagnosis of invasive pulmonary Aspergillosis in patients with hematological malignancies. J Infect.

[20] Buderer NMF (1996). Statistical methodology: I. incorporating the prevalence of disease into the sample size calculation for sensitivity and specificity. Acad Emerg Med..

[21] Jenks JD, Mehta SR, Taplitz R, Aslam S, Reed SL, Hoenigl M (2019). Point-of-care diagnosis of invasive aspergillosis in non-neutropenic patients: Aspergillus Galactomannan Lateral Flow Assay versus Aspergillus-specific Lateral Flow Device test in bronchoalveolar lavage. Mycoses.

[22] Maertens J, Maertens V, Theunissen K, Meersseman W, Meersseman P, Meers S (2009). Bronchoalveolar lavage fluid galactomannan for the diagnosis of invasive pulmonary aspergillosis in patients with hematologic diseases. Clin Infect Dis.

[23] Donnelly JP, Chen SC, Kauffman CA, Steinbach WJ, Baddley JW, Verweij PE (2019). Revision and update of the consensus definitions of invasive fungal disease from the European organization for research and treatment of cancer and the mycoses study group education and research consortium. Clin Infect Dis..

[24] Verweij PE, Rijnders BJA, Brüggemann RJM, Azoulay E, Bassetti M, Blot S (2020). Review of influenza-associated pulmonary aspergillosis in ICU patients and proposal for a case definition: an expert opinion. Intensive Care Med..

